# Supplementary material for: Treatment success for patients with tuberculosis receiving care in areas severely affected by Hurricane Matthew – Haiti, 2016
Source: PLoS One. 2021 Mar 17;16(3):e0247750. doi: 10.1371/journal.pone.0247750 (PMC7968710; doi:10.1371/journal.pone.0247750)
Supplement: S1 Appendix — (DOCX) [file pone.0247750.s001.docx]

**Assessment of TB facilities and services after the hurricane**

**Health Facility: _______________________________________Date________________**

**Capacity before the hurricane**

**Screening**

| 1. Total number of patients evaluated 6 months before the hurricane: |  |
| --- | --- |
| 2. Total number of patients diagnosed with TB 6 months before the hurricane: |  |

**Treatment**

| 3. Total number of patients who started anti-TB treatment 6 months before the hurricane: |  |
| --- | --- |
| 4. Total number of patients on treatment 6 months before the hurricane: |  |
| 5. Number of health care providers working at the facility before the hurricane: |  |
| 6. Was microscopy routinely available at the health facility? | 🞎 Yes 🞎 No |
| 7. TB drugs were routinely available at the facility before the hurricane: | RHEZ__________ RH___________ |

**Capacity after the hurricane**

**TB services**

8. How long were TB services interrupted after the hurricane?

🞎 No interruption 🞎 Less than 1 week 🞎 1-2 weeks 🞎 more than 2 weeks, specify:_____weeks

**Infrastructure and materials**

9. Status of building: 🞎 Intact 🞎 Partially damaged 🞎 Destroyed

10. Microscope status:  🞎 Functional 🞎 Damaged 🞎 Lost

11. Status of symptoms register: 🞎 Intact 🞎 Damaged 🞎 Lost

12. Status of laboratory register: 🞎 Intact 🞎 Damaged 🞎 Lost

13. Status of TB case register: 🞎 Intact 🞎 Damaged 🞎 Lost

14. Status of TB treatment cards: 🞎 Intact 🞎 Damaged 🞎 Lost

**Screening**

| 15. Total number of patients evaluated for TB since the hurricane: |  |
| --- | --- |
| 16. Have there been fewer patients evaluated for TB since the hurricane? | 🞎 Yes 🞎 No |
| 17. If yes, why? | __TB drugs not available  __Damaged facility  __Patients cannot reach facility  __No microscopy  __Other, specify______________  __Unknown reason |
| 18. Total number of new patients diagnosed with TB since the hurricane: |  |
| 19. Have there been fewer patients diagnosed with TB since the hurricane? | 🞎 Yes 🞎 No |
| 20. If yes, why? | __TB drugs not available  __Damaged facility  __Patients cannot reach facility  __Other, specify___________  __Unknown reason |

**Treatment**

| 21. Total number of new patients who started anti-TB treatment since the hurricane: |  |
| --- | --- |
| 22. Have there been fewer patients diagnosed with TB since the hurricane? | 🞎 Yes 🞎 No |
| 23. If yes, why? | __TB drugs not available  __Damaged facility  __Patients cannot reach facility  __Other, specify_______________  __Unknown reason |
| 24. Total number of patients receiving anti-TB treatment at the facility: |  |
| 25. Have there been fewer patients receiving anti-TB treatment at the facility since the hurricane? | 🞎 Yes 🞎 No |
| 26. If yes, why? | __TB drugs not available  __Damaged facility  __Patients cannot reach the facility  __Providers cannot reach the facility  __Other, specify_______________  __Unknown reason |

**Health care providers**

| 27. Total number of health care providers working at the facility since the hurricane: |  |
| --- | --- |
| 28. Have there been fewer health care providers working at the health facility since the hurricane? | 🞎 Yes 🞎 No |
| 29. If yes, why? | __Provider(s) hurt during hurricane  __Provider(s) died during hurricane  __Lack of transport  __Damaged personal residence  __Other, specify________________  __Unknown reason |
| 30. TB drugs available since the hurricane: | RHEZ__________ RH___________ |
| 31. Have there been fewer TB drugs since the hurricane? | 🞎 Yes 🞎 No |
| 32. If yes, why? | __Damaged warehouse  __Provider(s) hurt during the hurricane  __Provider(s) died during hurricane  __Lack of transport  __Provider’s home was damaged  __Other, specify_________________  __Unknown reason |
