# Supplementary material for: Treatment success for patients with tuberculosis receiving care in areas severely affected by Hurricane Matthew – Haiti, 2016
Source: PLoS One. 2021 Mar 17;16(3):e0247750. doi: 10.1371/journal.pone.0247750 (PMC7968710; doi:10.1371/journal.pone.0247750)
Supplement: S2 Appendix — (DOCX) [file pone.0247750.s002.docx]

**Post-disaster TB Patient Tracking Form**

Name of TB Health Facility: _________________________________________________

**Patient information**

- Name:_________________________________ Age:__________ Gender: 🞎M 🞎F
- Address and telephone number on file:________________________________________
- Smear status: 🞎Positive 🞎Negative
- Treatment category: 🞎New 🞎Retreatment
- Treatment start date_____________
- Last medication distribution date:____________
- Number of days of medication given at last visit: ____________
- TB regimen: 🞎RHEZ 🞎Other, specify: ________________
- Whether contact tracing already performed: 🞎Yes 🞎No

**Patient tracking**

- Status of clinic: 🞎Functional 🞎Not functional
- What effort has been made to locate the patient? 🞎Patient came to clinic 🞎Telephone call 🞎Home visit 🞎Not yet attempted 🞎Other, specify:______________________
- Outcome of patient tracking: 🞎Located 🞎Not located

**For located patients**

- Date patient located:____________________
- State address: 🞎Same 🞎Other, specify:________________________________________
- Physical status of the patient: 🞎Uninjured 🞎Injured 🞎Dead
- Current symptoms: 🞎None 🞎Fever 🞎Cough 🞎Night sweats 🞎Weight loss
- Number of days of medication missed since the event: ___________
- Meds for # days given: ___________
- Plan for ensuring ongoing treatment:

🞎 Continuing care at the same clinic

🞎 Instructed to visit another clinic, specify: ___________________________________

🞎 Not able to travel

**Facility name:___________________________________**

**Date: _______________**

|  | **Last name** | **First name** | **Treatment start date** | **Last visit date** | **Was patient tracking form completed? (Yes/No)** | **Was patient located? (Yes/No)** | **Date patient located** |
| --- | --- | --- | --- | --- | --- | --- | --- |
| 1 |  |  |  |  |  |  |  |
| 2 |  |  |  |  |  |  |  |
| 3 |  |  |  |  |  |  |  |
| 4 |  |  |  |  |  |  |  |
| 5 |  |  |  |  |  |  |  |
| 6 |  |  |  |  |  |  |  |
| 7 |  |  |  |  |  |  |  |
| 8 |  |  |  |  |  |  |  |
| 9 |  |  |  |  |  |  |  |
| 10 |  |  |  |  |  |  |  |
| 11 |  |  |  |  |  |  |  |
| 12 |  |  |  |  |  |  |  |
| 13 |  |  |  |  |  |  |  |
| 14 |  |  |  |  |  |  |  |
| 15 |  |  |  |  |  |  |  |
| 16 |  |  |  |  |  |  |  |
| 17 |  |  |  |  |  |  |  |
| 18 |  |  |  |  |  |  |  |
| 19 |  |  |  |  |  |  |  |
| 20 |  |  |  |  |  |  |  |

**List of patients receiving TB treatment before the Disaster**
